# Inhibitory effects of ZSTK474, a novel phosphoinositide 3-kinase inhibitor, on osteoclasts and collagen-induced arthritis in mice

**DOI:** 10.1186/ar3019

**Published:** 2010-05-18

**Authors:** Shoko Toyama, Naoto Tamura, Kazuhiko Haruta, Takeo Karakida, Shigeyuki Mori, Tetsuo Watanabe, Takao Yamori, Yoshinari Takasaki

**Affiliations:** 1 Department of Internal Medicine and Rheumatology, Juntendo University School of Medicine, 2-1-1 Hongo, Bunkyo-ku, Tokyo, 113-8421, Japan; 2 Central Research Laboratory, Zenyaku Kogyo Co., Ltd, 2-33-7 Oizumimachi, Nerima-ku, Tokyo, 178-0062, Japan; 3 Department of Biochemistry, School of Dental Medicine, Tsurumi University, 2-1-3 Tsurumi, Tsurumi-ku, Yokohama, Kanagawa, 230-8501, Japan; 4 Division of Molecular Pharmacology, Cancer Chemotherapy Center, Japanese Foundation for Cancer Research, 3-10-6 Ariake, Koto-ku, Tokyo, 135-8550, Japan

## Abstract

**Introduction:**

Targeting joint destruction induced by osteoclasts (OCs) is critical for management of patients with rheumatoid arthritis (RA). Since phosphoinositide 3-kinase (PI3-K) plays a critical role in osteoclastogenesis and bone resorption, we examined the effects of ZSTK474, a novel phosphoinositide 3-kinase (PI3-K)-specific inhibitor, on murine OCs *in vitro *and *in vivo*.

**Methods:**

The inhibitory effect of ZSTK474 on OC formation was determined and compared with other PI3-K inhibitors by counting tartrate-resistant acid phosphatase (TRAP)-positive multinucleated cells after culturing murine bone marrow monocytic OC precursors, and RAW264.7 cells. Activation of Akt and expression of nuclear factor of activated T cells (NFAT) c1 in cultured RAW264.7 cells were examined. The suppressing effect of ZSTK474 on bone resorption was assessed by the pit formation assay. The *in vivo *effects of ZSTK474 were studied in collagen-induced arthritis (CIA) in the mouse. Oral daily administration of ZSTK474 was started either when more than half or when all mice developed arthritis. Effects of ZSTK474 were evaluated using the arthritis score and histological score of the hind paws.

**Results:**

ZSTK474 inhibited the differentiation of bone marrow OC precursors and RAW264.7 cells in a dose-dependent manner. The inhibitory effect of ZSTK474 was much stronger than that of LY294002, the most commonly used PI3-K inhibitor. In addition, ZSTK474 suppressed the bone resorbing activity of mature OCs. Moreover, oral daily administration of ZSTK474, even when begun after the development of arthritis, ameliorated CIA in mice without apparent toxicity. Histological examination of the hind paw demonstrated noticeable reduction of inflammation and of cartilage destruction in ZSTK474-treated mice. ZSTK474 also significantly decreased OC formation adjacent to the tarsal bone of the hind paw.

**Conclusions:**

These findings suggest that inhibition of PI3-K with ZSTK474 may potentially suppress synovial inflammation and bone destruction in patients with RA.

## Introduction

Rheumatoid arthritis (RA) is a systemic autoimmune disease characterized by chronic inflammation of the synovium as well as by destruction of inflamed joints through bone erosion. The management of patients with RA consists of both reduction of inflammation and protection of the joints from structural damage [1]. Some anti-rheumatic drugs, including biologics, are quite useful but are not effective in all patients; hence, new therapeutic agents are required.

It has been speculated that joint destruction is directly caused by osteoclasts (OCs) [2], which differentiate from monocytic precursors that have infiltrated the inflamed joints. After this infiltration, monocytic precursors convert to tartrate -resistant acid phosphatase (TRAP)-positive cells and fuse with each other, eventually forming giant multinucleated OCs. Although the growth and differentiation of OCs mainly depend on receptor activator of nuclear factor κB ligand (RANKL) and macrophage-colony stimulating factor (M-CSF), proinflammatory cytokines, such as tumor necrosis factor (TNF)-α, which are over-expressed in the inflamed joints, promote this process [3]. After differentiation, ανβ3 integrins on differentiated OCs engage with the bone extracellular matrix; this process is followed by bone resorption [4,5]. It has been demonstrated that this increased resorbing activity of OCs results not only in bone erosion and further joint destruction but also in systemic osteoporosis in patients with RA. Therefore, suppressing OCs is a major aspect of RA therapy [6,7].

Signal transduction via the phosphoinositide 3-kinase (PI3-K)/Akt pathway is essential for regulating cellular responses, such as proliferation, survival, migration, motility and tumorigenesis, in a variety of cell types [8], not just OCs. Class I PI3-Ks are heterodimers and are found in four isoforms. Class IA PI3-Ks (PI3-Kα, PI3-Kβ and PI3-Kδ) are composed of a catalytic subunit p110 (α, β, or δ) and a regulatory subunit p85 (α or β), and activated through tyrosine kinase signaling. The class IB PI3-K (PI3-Kγ) is a heterodimer consisting of a catalytic subunit p110γ associated with one of two regulatory subunits, p101 and p84, and activated via seven-transmembrane G-protein-coupled receptors (GPCRs) [9]. Whereas the expression of PI3-Kα and PI3-Kβ is ubiquitous, that of PI3-Kδ and PI3-Kγ is mainly restricted to hematopoietic cells [8].

Many signal transduction molecules are involved in different phases of growth and development in OCs, such as Src homology-2 (SH2)-containing inositol-5-phosphatase (SHIP), Vav3, Gab2, extracellular signal-regulated kinase (ERK) and p38 mitogen-activated protein kinase (MAPK) [10-14]. In OCs, PI3-K is a major downstream effecter of the M-CSF receptor, RANK, and αβν3 integrin. The importance of PI3-K for differentiation, survival and motility of OCs has been demonstrated by using the PI3-K inhibitors wortmannin and LY294002 [15-22], and also by studying mice deficient in the expression of the p85α subunit of class IA PI3-K [23]. In addition, several transcription factors, including NF-kB, c-fos, AP-1, PU.1, and CREB, are involved in regulating osteoclastogenesis in its early or late phase, and expression of NFATc1 is specific to the RANKL induced-signaling pathway and essential for terminal differentiation of OCs [24,25].

Wortmannin and LY294002, potent inhibitors of PI3-K that have been extensively used for studying *ex vivo *PI3-K-driven signal pathways, also inhibit other related enzymes [9,26]. LY294002 causes severe dermal toxicity [27], and wortmannin and its analog has shown hepatic toxicity [28] when administered in mice. ZSTK474, a synthesized *s*-triazine derivative that strongly inhibited the growth of tumor cells, was subsequently identified as a novel PI3-K-specific inhibitor [29-33]. Furthermore, ZSTK474 is suitable for oral administration, and demonstrated marked *in vivo *antitumor activity in mice grafted with human cancer cells without showing toxicity to major organs [29].

Since the action of ZSTK474 on OCs is unknown, we examined the effects of ZSTK474 in an *in vitro *OC culture system and found strong inhibitory effects on the differentiation and bone resorbing activity of OCs. Moreover, daily administration of ZSTK474 ameliorated collagen-induced arthritis (CIA) in mice, remarkably reducing the migration of inflammatory cells and OCs in the synovial tissue.

## Materials and methods

### PI3-K inhibitors

ZSTK474 and IC87114 (a PI3-Kδ-selective inhibitor) were synthesized at Central Research Laboratories of Zenyaku Kogyo Co. Ltd. (Tokyo Japan). LY294002 was purchased from Sigma Chemical Co. (St Louis, MO, USA). AS605240 (a PI3-Kδ-selective inhibitor) was purchased from Calbiochem (Schwalbach, Germany). In *in vivo *experiments, ZSTK474 was prepared as a solid dispersion [34].

### Animals

Male DBA/1 mice (eight weeks old) were purchased from Charles River Laboratories Japan (Kanagawa, Japan). They were maintained at approximately 22°C with a 12-hour light/dark cycle and given standard chow and tap water *ad libitum*. Newborn ddY mice were obtained from the Japan SLC, Inc. (Shizuoka, Japan). All animal experiments were approved by the local ethical committees of each institution.

### Osteloclast formation

*In vitro *OC formation was examined as previously described [35]. Briefly, primary osteoblasts derived from growing calvarial cells of newborn ddY mice at three- to four-days of age were suspended in alpha-minimum essential medium (α-MEM, Sigma) supplemented with 10% (v/v) fetal bovine serum (FBS, Gibco BRL, Gaithersburg, MD, USA), 100 U/ml penicillin and 100 μg/ml streptomycin, and plated at a density of 2 × 10^4 ^cells/well in 24-well plates (Corning Incorporated, Corning, NY, USA) overnight. Mouse bone marrow cells containing monocytic OC precursors were removed aseptically from the tibiae of four- to six-week old ddY male mice, and co-cultured on adherent osteoblasts at a density of 1.0 × 10^6^cells/well in medium containing 10^-7 ^M 1α,25-(OH)_2_D_3 _(Wako Pure Chemical Industries, Ltd., Osaka, Japan) for five to six days in the presence or absence of varying concentrations of ZSTK474 or other PI3-K inhibitors. Otherwise, non-adherent bone marrow cells were cultured alone with 10 ng/ml of M-CSF (R & D Systems, Minneapolis, MN, USA) for two days, and then adherent cells were cultured with 100 ng/ml of soluble RANKL (sRANKL) (R & D Systems) for three days. In some experiments, RAW264.7 cells (American Type Culture Collection, Manassas, VA, USA) were plated at a density of 2.5 × 10^4 ^cells/well in a 24-well tissue culture plate overnight, and sRANKL (100 ng/ml), TNF-α (50 ng/ml) and ZSTK474 were added. The medium was changed every two to three days. The cells were fixed with 3.7% formalin, permeabilized with 0.1% Triton X-100, and stained with TRAP. OC formation was determined by counting TRAP-positive multinucleated cells having three or more nuclei, and OCs were counted in each set of duplicated wells.

### Real time-polymerase chain reaction (PCR) for the quantification of RANKL expression

The osteoblasts were plated at a density of 2 × 10^5 ^cells/well in six-well plates, and cultured with or without 1α,25-(OH)_2_D_3 _for 24 hours in the presence or absence of ZSTK474. Total RNA was extracted using a total RNA isolation kit (Ambion Inc., Austin. TX, USA), and 3 μg of the total RNA was reverse transcribed using a You-prime Fast-Strand Breads kit (Amersham Pharmacia Biotech, Inc., Piscataway, NJ, USA). The primers used in PCR were 5'-GACTCGACTCTGGAGAGT-3' (sense primer) and 5'-GAGAACTTGGGATTTTGATGC-3' (antisense primer) for RANKL and 5'-AGCCATGTACGTAGCCATCC (sense primer) and 3'-CTCTCAGCTGTGGTGGTGAA (antisense primer) for β-actin. Real-time PCR was performed using 1 μg of cDNA and Power SYBR Green Master Mix (Applied Bio Systems, Foster City, CA, USA) on an ABI PRISM 7500 Sequence Detection System (Applied Bio Systems) with conditions at 95°C for 10 minutes, followed by 40 cycles at 95°C for 15 seconds and 60°C for one minute. The expression of RANKL was quantified using the comparative C_T_, applying the formula X_n _= 2^-ΔCT^, where X_n _is the relative amount of target gene in question and ΔC_T _is the difference between the C_T _of the house keeping gene for a given sample [36].

### Western blotting for Akt and NFATc1

RAW264.7 cells were plated at a density of 2.5 × 10^5 ^cells/well in a six-well tissue culture plate overnight, and ZSTK474 was added. After incubation for 30 minutes, 50 to 100 ng/ml of sRANKL, or sRANKL plus TNF-α (50 ng/ml), was added and the cells were incubated for the indicated time. Cells were washed twice with ice-cold phosphate-buffered saline (PBS) containing 1% phosphatase inhibitor cocktail (Sigma), detached with a cell scraper, centrifuged, and lysed with lysis buffer (1% Triton X-100, 1% phosphatase inhibitor cocktail and 1 mM of PMSF in Tris-buffer, pH 7.6). The lysates were boiled with sodium dodecyl sulfate (SDS) -sample buffer and run on SDS-PAGE followed by blotting with a 1:1000 dilution of anti-phospholylated Akt (anti-phospho Akt), anti-Akt, anti-IκB, anti-phospho cJun, anti-phospho p42/p44, anti-β-actin (Cell Signal Technology, Inc., Beverly, MA, USA) and anti-NFAT1c monoclonal antibody (Santa Cruz Biotechnology, Santa Cruz, CA, USA).

### Immunofluorescence microscopy

RAW264.7 cells (200 μl, 2.5 × 10^5^/ml) were plated onto Lab Tek Chamber slide (Thermo Fisher Scientific, Rochester, NY, USA) overnight. After treatment with 0.1 μM of ZSTK474 for 30 minutes, 100 ng/ml of sRANKL and 50 mg/ml of TNF-α were added, and the cells were cultured for 48 hours. Then, the cells were fixed with 4% paraformaldehyde, washed with PBS three times, permeabilized with 0.1% Triton X-100 in PBS, and blocked with 10% normal goat serum (Nichrei, Tokyo, Japan). The cells were incubated with anti-NFATc1 antibody diluted in PBS (1:50) for one hour, washed with PBS, and followed with phycoerythrin-conjugated goat anti-rabbit IgM+IgG (H+L chain specific, Beckman Coulter) for another one hour. The cells were postfixed in Aqua-Poly/Mount (Polysciences, Washington, PA, USA) and viewed using fluorescence microscope (Nikon ECLIPSE E600/Y-FL).

### Bone resorbing activity of OC

A 10 cm culture dish (Corning) was coated with 5 ml of type I collagen mixture at 4°C. The dish was placed in a CO_2 _incubator at 37°C for 10 minutes to render the aqueous type I collagen gelatinous. Primary osteoblasts (5 × 10^5 ^cells/dish) and bone marrow cells (6 × 10^6 ^cells/dish) were co-cultured on the collagen gel-coated dish for five days. The dish was then treated with 4 ml of 0.2% collagenase solution (Nitta Gelatin Co., Osaka, Japan) for 20 minutes at 37°C in a shaking water bath (60 cycles/minute). The cells were collected by centrifugation at 600 rpm for three minutes, then washed and suspended with α-MEM containing 10% FBS (OC preparation). Dentine slices (Immunodiagnostic Systems, Ltd., Boldon, UK) were cleaned by ultrasonication in distilled water, sterilized using 70% ethanol, dried under ultraviolet light, and placed in 96-well plates. A 0.1-ml aliquot of the OC preparation was transferred onto the slices. After incubation for 72 hours in the presence or absence of the PI3-K inhibitors, the medium was removed and 1 ml of 1 M NH_4_OH was added to each well and incubated for 30 minutes. The dentin slices were then cleaned by ultrasonication, stained with hematoxylin (Wako Pure Chemical Industries) for 35 to 45 seconds, and washed with distilled water. The area of resorption pits that formed on dentine slices was observed under a light microscope and measured.

### CIA in mice

Male DBA/1 mice, eight-weeks of age, were injected intradermally in the base of the tail with 200 μg of bovine type II collagen (Collagen Gijutsu Kenshu-Kai., Tokyo, Japan) emulsified in complete Freund's adjuvant (Difco, Detroit, MI, USA) on Day 1, and the same amount of the antigen emulsified in incomplete Freund's adjuvant (Difco) on Day 22. When half of the mice had developed arthritis (Day 28), the mice were randomly divided into four groups of eight mice. Each group orally received vehicle or 25, 50, 100 mg/kg of ZSTK474, once/day. In another therapeutic protocol, 100 mg/kg of ZSTK474 was administered from the day when all mice developed arthritis (Day 31). Total arthritis score was defined as the sum of the paw swelling scores for each paw (0 to 4 per paw), with a maximum score of 16. In the semi-therapeutic protocol, the mice were killed on Day 50, and the right hind paws were removed, fixed in paraformaldehyde, decalcified in Kalkitox (Wako Pure Chemical Industries, Ltd.), embedded in paraffin and sectioned. The sections were then stained with hematoxylin and eosin (H&E) or safranin O to assess hyperplasia of synovial tissue, infiltration of leukocytes, and destruction of cartilage. Each parameter was graded separately and assigned a severity score as follows: grade 0, no detectable change: 1 to 4, slight to severe changes. The number of OC in talus was counted in every third 6 μm section. To examine *in vivo *OC formation in CIA mice, the hind paws were removed on Day 52 and rapidly frozen in the therapeutic protocol. The frozen tissue was sectioned according to the method described previously [37] and the sections were stained with H&E or TRAP. Plasma TRACP5b levels were measured using a mouse TRAP™ Assay (Immunodiagnostic System Ltd).

### Statistical analysis

Statistical significance of differences was assessed by one-way analysis of variance (ANOVA) followed by Dunnett's test or the Student's *t*-test for comparison of two samples. Statistical tests were performed using Kaleida graph 3.6 (Synergy Software, Reading, PA, USA). In all analyses, *P *< 0.05 was considered statistically significant.

## Results

### Inhibitory effects of ZSTK474 on OC formation in co-culture system

**Figure 1:**
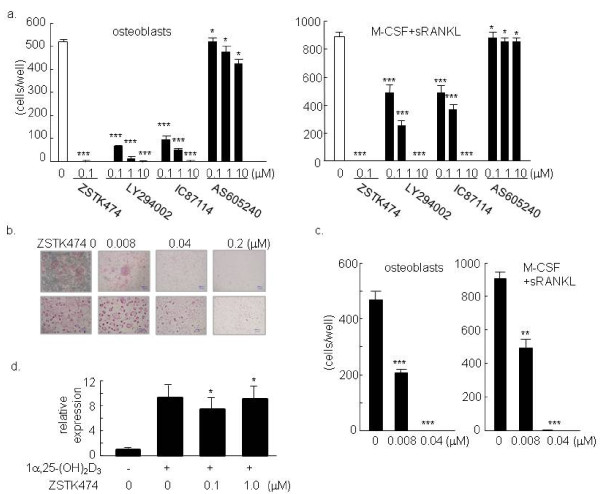
**Inhibitory effect of ZSTK474 on OC formation**. Mouse bone marrow cells containing OC precursors were cultured with osteoblasts in the presence of 1α,25-(OH)_2_D_3 _for five days. Indicated concentrations of ZSTK474, LY294002, AS605240 (a PI3-Kγ-selective inhibitor), or IC87114 (a PI3-Kδ-selective inhibitor) were added at the initiation of cultures. Mouse bone marrow cells were also cultured with M-CSF (10 ng/ml) for two days and then with M-CSF and sRANKL (100 ng/ml) for another three days. The inhibitors were added simultaneously with sRANKL. **a**) and **c) **TRAP-positive multinucleated cells were counted as OC. The columns and bars indicate the mean and standard deviation (S.D.) from duplicated wells. **b) **OC formation in co-culture with osteoblast (upper) and culture with M-CSF and sRANKL (lower). Representative results were shown in a, b, and c. **d**) RANKL mRNA was measured using real-time PCR, with results normalized the value of β-actin. The columns and bars indicate the mean and S.D. of three independent experiments. * = P > 0.05, ** = P < 0.01, *** = P < 0.001.

To determine whether ZSTK474 could inhibit osteoclastogenesis *in vitro*, mouse bone marrow monocytic precursors were co-cultured with osteoblasts together with 1α,25-(OH)_2_D_3 _in the presence or absence of various concentrations of ZSTK474 or other PI3-K inhibitors. The effect was also examined in OC differentiation of the bone marrow precursors in response to M-CSF and sRANKL. OC formation was significantly inhibited by ZSTK474 in both culture systems, and this inhibitory effect was much stronger than that of LY294002 (Figure 1a), the most commonly used PI3-K inhibitor at present. IC87114 also inhibited OC formation similarly to LY294002, whereas AS605240 had virtually no effect on the OC differentiation, indicating that PI3-Kδ might play a more important role in OC formation in these culture systems. ZSTK474 suppressed OC formation in a dose-dependent manner at lower concentrations (Figure 1b and 1c). No TRAP-positive cells were observed with 0.2 μM of ZSTK474, suggesting that differentiation of OCs was completely suppressed at this concentration. On the other hand, 0.04 μM of ZSTK474 were likely to allow the monocytic precursors to differentiate into small TRAP-positive cells, but not to form large OCs (Figure 1b). In addition, ZSTK474, even at 1 μM, did not decrease the expression of RANKL mRNA in osteoblasts cultured with 1α,25-(OH)_2_D_3 _(Figure 1d), indicating that RANKL expression on osteoblasts might not be involved in suppressing effect of ZSTK474 on OC differentiation.

### Inhibition of Akt phosphorylation and NFATc1 expression in RAW264.7 cells by ZSTK474

**Figure 2:**
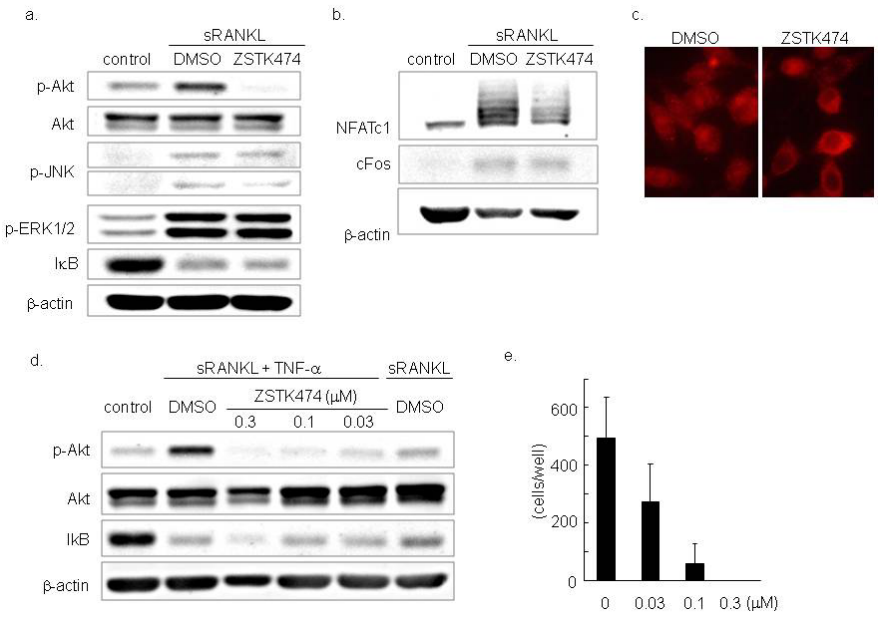
**Suppressive effect of ZSTK474 on OC precursor cells**. **a) **Inhibition of Akt phosphorylation by ZSTK474. RAW264.7 cells were incubated with or without 0.1 μM of ZSTK474 for 30 minutes and for another 15 minutes in the presence of soluble RANKL. The phosphorylated Alt (p-Akt) and whole Akt (Akt) were examined by the Western blotting. **b) **The expression of NFATc1 was determined in RAW264.7 cells cultured for 48 hours in the presence of soluble RANKL with or without ZSTK474 pretreatment. **c) **NFATc1 localization was visualized using immunofluorescence microscopy in RAW264.7 cells cultured with sRANKL and TNF-α for 24 hours. Treated with vehicle (left) and 0.1 μM of ZSTK474 (right). **d) **The phosphorylation of Akt in RAW264.7 cells cultured with soluble RANKL and TNF-α was inhibited by ZSTK474. **e) **RAW264.7 cells were cultured in the presence of RANKL and TNF-α in the presence or absence of ZSTK474. The number of TRAP staining-positive multinucleated cells was counted.

To confirm that ZSTK474 affected the monocytic precursors but not the osteoblasts, we examined its effect on the phosphorylation of Akt in RAW264.7 cells. Phosphorylation of Akt induced by sRANKL (100 ng/ml) was abolished by ZSTK474 (Figure 2a). However, ZSTK474 did not inhibit the degradation of IκB and phosophorylation of JNK and ERK1/2 induced by sRANKL. On the other hand, the expression of NFATc1, which occurs in the late phase of OC differentiation and promotes terminal osteoclastogenesis in association with a complex of cJun and cFos [38,39], was attenuated in RAW264.7 cells treated with sRANKL by 0.1 μM of ZSTK474, although ZSTK474 did not apparently affect the expression of cFos (Figure 2b). We further analyzed translocation of NFATc1 by immunofluorescence microscopy. Calcium entry to OC precursor cells activates the calcium/calmodulin-dependent pathway, leading to NFATc1 translocation into the nucleus. ZSTK474 repressed the translocation of NFATc1 to the nucleus in response to sRANKL and TNF-α (Figure 2c). These results indicated that ZSTK474 at least blocked the RANK/RANKL-PI3-K/Akt cascade in monocytic precursors, resulting in inhibition of OC differentiation.

### Inhibitory effects of ZSTK474 on OC formation induced by both RANKL and TNF-α

We next examined the effects of ZSTK474 on OC formation induced by RANKL and TNF-α, since it was speculated that TNF-α enhanced OC formation in RA. In fact, RANKL-induced phosphorylation of Akt was enhanced by the addition of TNF-α (Figure 2d). ZSTK474 (0.03, 0.1, and 0.3 μM) inhibited the phosphorylation of Akt induced by RANKL (100 ng/ml) and TNF-α (50 ng/ml) in RAW264.7 cells (Figure 2d). Moreover, the OC formation induced by RANKL (100 ng/ml) and TNF-α (50 ng/ml) was inhibited by ZSTK474 in a dose-dependent manner. OC formation was completely inhibited by ZSTK474 (0.3 μM, Figure 2e).

### Inhibition of bone resorbing activity of OC by ZSTK474

**Figure 3:**
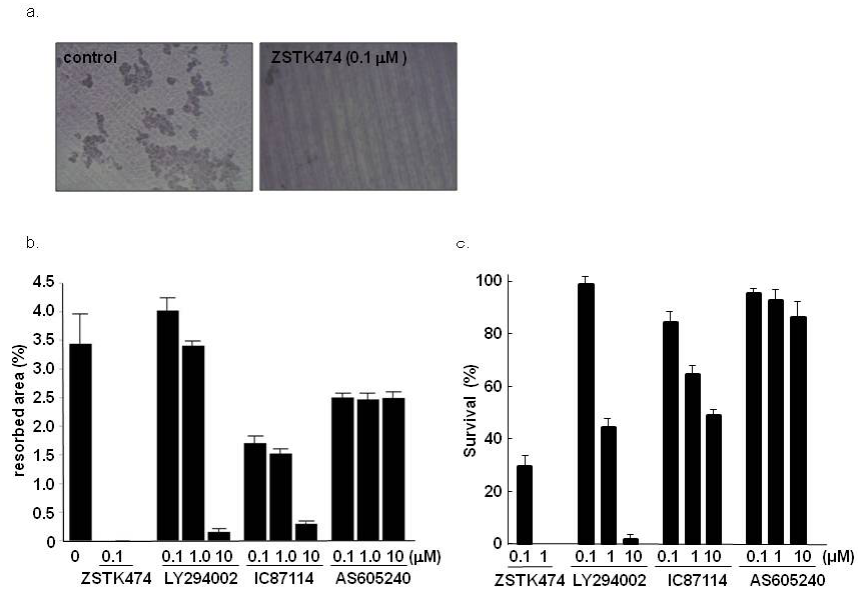
**Blocking bone resorbing activity of OCs with ZSTK474**. Mouse bone marrow derived monocytic OC precursors were co-cultured with osteoblasts in the presence of 1α,25-(OH)_2_D_3 _on a collagen gel-coated dish. The matured OCs were collected and transferred onto dentin slices and incubated for 72 hours in the presence or absence of ZSTK474 and other PI3-K inhibitors. The dentin slices were stained with hematoxylin, and the pits formed in the resorbed area on the slices were observed **(a) **and measured **(b) **under a light microscope. (**c) **Matured OCs, differentiated from bone marrow cells as described above, were further cultured with 5 ng/ml of TNF-α and the PI3-K inhibitors. After 24 hours, TRAP-positive multinucleated cells were counted, and the percentages of surviving cells were calculated. The columns and bars indicate the mean and S.D. from duplicated wells in **a) **and **c)**.

We next examined whether ZSTK474 also inhibited the bone-resorbing activity of mature OCs. The OCs that had matured on the collagen-gel were transferred onto dentine slices, the total areas of the resorbed pits were measured after three days culture. This experiment revealed that 0.1 μM of ZSTK474 completely prevented pit formation by OCs (Figure 3a, b). LY294002 and IC87114, but not AS605240, also inhibited the bone resorption more weakly (Figure 3b). Because PI3-K is important for OC survival [19], it was supposed that PI3-K inhibited the survival of mature OCs and consequently suppressed the bone resorption. Therefore, we tested whether ZSTK474 affected the survival of mature OCs. Complete and partial inhibition of OC survival was observed in the presence of 1 μM and 0.1 μM of ZSTK474, respectively (Figure 3c).

### Amelioration of CIA in mice with oral administration of ZSTK474

**Figure 4:**
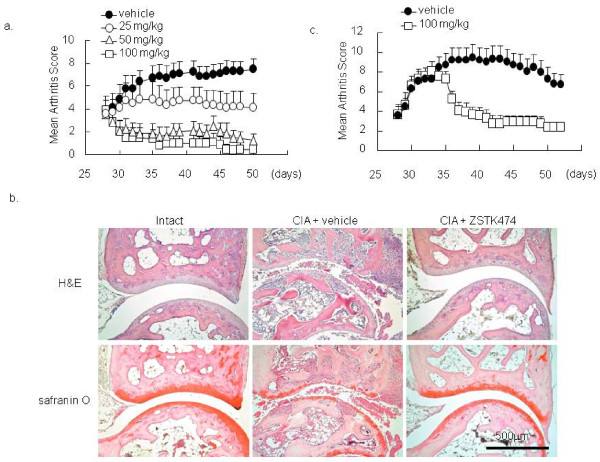
**Oral administration of ZSTK474 ameliorated CIA in mice**. *In vitro *effect of ZSTK474 was examined in mice CIA. On Day 28, when half of the mice had developed arthritis, oral administration of ZSTK474 was commenced once a day. **a) **Arthritis scores were compared among the groups. **b) **Synovial tissues from the hindpaws of vehicle-treated CIA mice, 50 mg ZSTK474-treated CIA mice and normal age-matched DBA mice were stained with hematoxylin and eosin (H&E) or with safranin O. Representative results are shown. **c) **In the therapeutic protocol, 100 mg/kg of ZSTK474 was started on Day 31, when all mice had developed arthritis.

**Figure 5:**
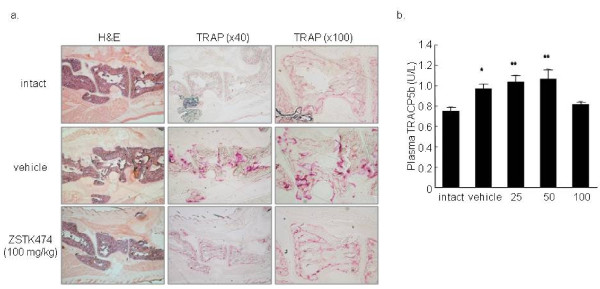
**Administration of ZSTK474 inhibited *in vivo *OC formation and bone resorption in CIA mice**. **a) **The synovial sections described above were stained with H&E and also with TRAP to examine *in vivo *OC formation. Representative results are shown. **b) **Plasma levels of TRACP5b were measured. The levels of TRACP5b in vehicle- 25 mg/kg, and 50 mg/kg ZSTK474-treated mice, but not 100 mg/kg ZSTK474-treated mice, were significantly raised in comparison with that of intact mice (*P < 0.05, **P < 0.01).

To determine whether interference with PI3-K activity by ZSTK474 reduces joint destruction *in vivo*, we examined the effects of ZSTK474 on CIA in mice. ZSTK474 was administered from the day when more than 50% of the mice developed arthritis (Day 28). While vehicle-treated mice developed active arthritis, administration of daily oral ZSTK474 ameliorated joint inflammation in a dose-dependent manner. The arthritis score reached 7.5 ± 0.9 by Day 50 in the vehicle-treated group, whereas oral administration of ZSTK474 reduced the arthritis score to 4.1 ± 1.2 (25 mg/kg, *P *< 0.05), 1.3 ± 0.6 (50 mg/kg, *P *< 0.001), and 0.5 ± 0.5 (100 mg/kg, *P *< 0.001, Figure 4a). Histological staining of the affected synovial tissues demonstrated that administration of ZSTK474 (50 mg/kg) markedly attenuated infiltration of inflammatory cells, proliferation of synovial fibroblasts and cartilage/bone destruction (Figure 4b, Table 1). Especially, the number of OCs in talus decreased significantly in ZSTK474 (50 mg/kg)-treated group (Table 1). Furthermore, a remarkable reduction was observed in the arthritis score even in the therapeutic protocol in which ZSTK474 administration was begun (100 mg/kg) after development of arthritis. At Day 52, there were highly significant differences between the vehicle-treated group and the ZSTK474 (100 mg/kg)-treated group (mean arthritis score: 6.8 ± 1.0 versus 2.4 ± 0.5, Figure 4c). TRAP-staining of the joint section confirmed numerous OCs adjacent to the tarsal bones of vehicle-treated mice, whereas TRAP-positive OC formation in ZSTK474-treated mice was markedly decreased (Figure 5a). In addition, plasma levels of TRACP5b, a biomarker of systemic bone resorption, raised significantly in vehicle-treated, 25 mg/kg, and 50 mg/kg ZSTK474-treated mice, compared to intact mice. In contrast, the TRACP5b levels were sustained in 100 mg/kg ZSTK474-treated mice (Figure 5b).

**Table 1:** Histological score and osteoclast number

	Synovium	Leukocyte	Cartilage/bone	Osteoclast
Vehicle (n = 6)	2.3 ± 0.8	1.7 ± 0.9	2.7 ± 0.6	62.0 ± 38.6
ZSTK474 (n = 6, 50 mg/kg)	0.0 ± 0.0	0.0 ± 0.0	0.8 ± 0.3	0.3 ± 0.2
*P*-value	0.009	0.073	0.036	0.024

## Discussion

In this study, we demonstrated that ZSTK474, a novel PI3-K-specific inhibitor, suppressed osteoclastogenesis and bone resorption. The *in vitro *inhibitory effect of ZSTK474 on OC formation, observed by culturing bone marrow cells, was much stronger than that of LY294002. Although both inhibit all isoforms of class I PI3-K, the inhibitory activities of ZSTK474 (IC_50_: PI3-Kα: 1.6 × 10^-8 ^M; PI3-Kβ: 4.4 × 10^-8 ^M; PI3-Kγ: 4.9 × 10^-8 ^M; PI3-Kδ: 4.6 × 10^-9 ^M) were much stronger than those of LY294002 (IC_50_: PI3-Kα: 5.5 × 10^-7 ^M; PI3-Kβ: 1.1 × 10^-5 ^M; PI3-Kγ: 1.2 × 10^-5 ^M; PI3-Kδ: 1.6 × 10^-6 ^M) on all isoforms, especially PI3-Kδ [30]. A PI3-Kδ-selective inhibitor, IC87114 (1 μM), completely inhibited OC formation, while a PI3-Kγ-selective inhibitor, AS605240, had no inhibitory effect on OC formation. These results indicate the involvement of PI3-Kδ in the OC culture system, consistent with a previous report which implicated a critical role of class IA PI3-K in OC formation by demonstrating that OC progenitor cells from mice lacking p85α, a regulatory subunit of class IA PI3-K, showed impaired growth and differentiation [23].

Blocking of the phosphorylation of Akt by ZSTK474 in RAW264.7 cells indicated that the inhibitory effect on OC formation observed in the bone marrow monocytic cells was due at least in part to suppression of PI3-K/Akt signal pathway in the OC precursors. This suggestion is supported by the observation that the consequent expression of NFATc1, an essential factor for terminal RANKL-induced differentiation of OCs [25,38], was also prevented by ZSTK474. The reduced expression of NFATc1 was dependent on neither NFkB nor cFos in the condition of this study. Additionally, translocation of NFATc1 into the nucleus was also inhibited by ZSTK474, implying that ZSTK474 might suppress the autoamplification, calcium-signal-mediated persistent activation [40], of NFATc1. Moreover, ZSTK474 inhibited the phosphorylation of Akt and OC differentiation induced by both RANKL and TNF-α, which are fundamental factors for OC formation in RA, implying that ZSTK474 might inhibit OC formation in patients with RA.

ZSTK474 also suppressed the bone resorbing activity of OCs as assessed in an *in vitro *pit formation assay. This could be explained by the inhibitory effect of ZSTK474 on survival of mature OCs in part. Likewise, signaling via PI3-K is crucial for remodeling and assembly of actin filaments, cell spreading and adhesion [41]. Furthermore, blocking PI3-K with ZSTK474 inhibited the membrane ruffling induced by platelet-derived growth factor (PDGF) in murine embryonic fibroblasts [29]. In OCs, the SH3 domain of tyrosine kinase, *c-src*, interacts with the p85 regulatory domain of PI3-K, and this signaling pathway is crucial for colony-stimulating factor-1-induced OC spreading [22]. Therefore, ZSTK474 might suppress the cytoskeletal change of OCs, resulting in the reduced bone resorption observed in this study.

ZSTK474 suppressed inflammation and also protected against joint destruction in CIA in mice. Although it is difficult to ascertain the direct effect of ZSTK474 on OCs in this model, the TRAP-staining of the synovial tissue sections demonstrated marked reduction of OC formation. In addition, plasma levels of TRACP5b, that reported to correspond with systemic but not localized bone resorption [42], were not increased in 100 mg/kg ZSTK474-treated mice. This result implied that 100 mg/kg of ZSTK474 possibly prevented the systemic bone resorption.

Both the semi-therapeutic and therapeutic treatments of ZSTK474 ameliorated joint inflammation in a mouse model of RA. This anti-rheumatic effect might be explained by contribution of PI3-K to activation, proliferation and migration of inflammatory cells, such as lymphocytes, macrophages, neutrophils, mast cells and synovial fibroblasts [9]. However, the titers of antibody to type II collagen were not significantly different between vehicle- and ZSTK474-treated mice in this experiment (data not shown). Regarding migration, chemokine receptors, such as the MCP-1 receptor and the RANTES receptor, are GPCRs that associate with PI3-Kγ and induce signals for chemotaxis of the inflammatory cells [9]. It was reported that the PI3-Kγ-selective inhibitor suppressed joint inflammation in mouse CIA by inhibiting migration of neutrophils to the joints [43]. This inhibitory process might occur in the ZSTK474-treated mice. Additionally, synovial pannus tissues of patients with RA express phosphorylated Akt [44] and exhibit tumor-like behaviors, such as angiogenesis, proliferation and invasion. A recent report demonstrated potent antiangiogenic activity for ZSTK474, which could be attributed to both inhibition of VEGF secretion by cancer cells and inhibition of PI3-K in endothelial cells [45]. These findings also account for the effects of ZSTK474 on CIA mice. In addition, ZSTK474 did not affect the count of peripheral white blood cells and red blood cells (data not shown). Further studies are underway to evaluate how ZSTK474 exerts anti-inflammatory activity *in vivo*.

Clinical studies have demonstrated that the degree of inflammation and the progression of joint destruction do not always correspond with each other [46,47]. In current therapy for RA, anti-rheumatic drugs are required not only to control the inflammation but also to suppress the joint destruction. On the other hand, recent reports have shown convincing pathogenic evidence for the involvement of class I PI3-K and Akt signaling pathways in synovial fibroblasts [44,48-52] and other cells [43,53,54] in patients with RA. Synovial tissue from patients with RA expressed higher levels of phosphorylated Akt than that from patients with osteoarthritis [44]. Moreover, blocking the PI3-K/Akt pathway by intracellular gene transfer of phosphatate and tensin homolog deleted on chromosome 10 (PTEN), which dephosphorylates phosphatidylinositol - 3,4,5 - tris - phosphate (Ptdlns(3,4,5)P_3_) and attenuates the downstream signals of PI3-K, CIA in rats [52]. Taken together, the present results indicate that PI3-K could be a potent target for RA therapy.

## Conclusions

We have demonstrated inhibitory effects of ZSTK474 on *in vitro *OC formations and CIA in mice. Inhibition of PI3-K with ZSTK474 may potentially have an anti-rheumatic effect in patients with RA.

## Abbreviations

CIA: collagen-induced arthritis; ERK: extracellular signal-regulated kinase; FBS: fetal bovine serum; GCPRs: G-protein-coupled receptors; MAPK: mitogen-activated protein kinase; M-CSF: macrophage-colony stimulating factor; NFATc1: nuclear factor of activated T cells c1; OCs: osteoclasts; PDGF: platelet-derived growth factor; PI3-K: phosphoinositide 3-kinase; PTEN: phosphatate and tensin homolog deleted chromosome 10; RA: rheumatoid arthritis; RANK: receptor activator of nuclear factor κB; RANKL: RANK ligand; SHIP: Src homology-2 (SH2)-containing inositol-5-phosphatase; TNF: tumor necrosis factor; TRAP: tartrate-resistant acid phosphatase; α-MEM: alpha-minimum essential medium

## Competing interests

KH, SM and TW were employed by Zenyaku Kogyo Co., Ltd (Tokyo, Japan), which is the proprietary company of ZSTK474. TY has a research fund from Zenyaku Kogyo Co., Ltd. ST, NT, TK and YT declare that they have no competing interests.

## Authors' contributions

ST performed data acquisition and was involved in drafting of the manuscript. NT contributed to the study design and did most of the drafting of the manuscript. KH designed the *in vivo *and part of the *in vitro *experiments, and carried out the analysis and interpretation of data; he was also involved in drafting of the manuscript. TK participated in the *in vitro *experiments and gave helpful advice. SM contributed essentially to the animal experiments. TW provided the synthesized PI3-K inhibitors used in this study. TY fundamentally participated in the concept of the study using ZSTK474. YT supervised conception and design of the study. All authors read and approved the final manuscript.
